# The Assessment of the Rationale for Urgent Head CT—Comparative Analysis of Referrals and Results of Examinations without and with Contrast Enhancement

**DOI:** 10.3390/medicina58101468

**Published:** 2022-10-16

**Authors:** Izabela Rosół, Jakub Ciesielka, Magdalena Matlakiewicz, Michał Grześków, Maciej Cebula, Katarzyna Gruszczyńska, Mateusz Winder

**Affiliations:** 1 Students’ Scientific Society, Department of Radiology and Nuclear Medicine, Medical University of Silesia, ul. Medyków 14, 40-752 Katowice, Poland; 2 Department of Radiology and Nuclear Medicine, Medical University of Silesia, ul. Medyków 14, 40-752 Katowice, Poland

**Keywords:** defensive medicine, computed tomography, head imaging, radiation, contrast enhancement

## Abstract

The study analyzes the correlation between the indications and results of head CT examinations in search of evidence of the excessive use of this diagnostic method. In total, 1160 referrals for urgent head CT were analyzed retrospectively, including the following parameters: patients’ sex and age, type of scan (C−, C+, angio-CT), description of symptoms and presence of diagnostic target. Pathologies identified by the radiologist were assigned to four classes, regarding the severity of diagnosed conditions. The analysis of the CT results has shown that over half (55.22%) of the examinations revealed no deviations or showed chronic, asymptomatic lesions. As many as 73.71% referrals constituted group 0 in terms of the lack of a diagnostic target of a specific pathology. The presence of specific clinical targeting in a referral correlated significantly with a higher frequency of acute diagnosis. Contrast-enhanced follow-up examinations allowed the unequivocal classification of patients into extreme classes (I or IV) and accurate identification of patients requiring urgent or chronic treatment. Excessive use of diagnostic imaging is harmful, not only to patients, who often are unnecessarily exposed to radiation, but also to the quality of healthcare, since it increases the costs and radiologists’ workload.

## 1. Introduction

Defensive medicine is defined as medicine practiced in such a way as to reduce the risk of litigation for medical malpractice, usually by over-performing diagnostic tests [1,2,3]. Defensive medicine not only negatively affects the quality of healthcare, but also potentially contradicts the main ethical principle in medicine: “primum non nocere” (Latin for “first do no harm”) [4,5,6,7]. This is especially true in the case of the excessive use of imaging examinations utilizing X-rays, such as radiography (X-ray) or computed tomography (CT) [8,9]. The phenomenon of “defense medicine” intensifies the profile of a patient with demanding behaviors, i.e., requesting diagnostic tests because of personal beliefs or information obtained from other patients or social media and the Internet [10].

The CT examination, thanks to its high resolution and accuracy in showing anatomical details, clearly excludes or confirms the vast majority of pathologies, becoming a powerful diagnostic tool, but it is also an overused test in “defense medicine”. The Polish atomic law states that radiodiagnostic procedures require a clear aim, have to be justified and the diagnostic benefit must exceed the possible health damages caused by the patients’ exposure to radiation. Following these rules is especially important as the total number of CT examinations performed in hospitals and by outpatient care providers worldwide is constantly growing every year [11,12,13]. According to the Organization for Economic Co-Operation and Development (OECD), the largest, more than three-fold increase was recorded in Korea between the years 2007 and 2019, where the annual number of CT examinations reached 74.7/1000 people and 242.8/1000 people, respectively.

The increase in the number of imaging examinations significantly affects radiologists’ workload, causes the development of occupational diseases, fatigue and stress and potentially increases the chance of errors [14,15,16,17,18]. It also has a direct negative impact on the patient’s health. It is estimated that 0.6–3% of cancers, especially thyroid and non-Hodgkin lymphomas in adults, and non-Hodgkin’s lymphomas and primary brain tumors in pediatric patients, are induced by radiation [19,20,21]. There are also reports showing other side effects of CT scans, such as an increased risk of cataracts or post-contrast complications [22,23]. The financial aspect of the growing number of imaging examinations and the increase in diagnostic costs, which disproportionately outweighs the potential benefits, should also be mentioned.

The aim of our work was to analyze the correlation between the number of performed CT scans of the head and the radiological results in the search for misuse of imaging examinations. Multiple studies have proven that this is a common problem, but few current reports exist on the above-mentioned issues in radiology [24,25,26]. In 2013, it was estimated that around a third of all CT and MRI scans worldwide were considered misused [8].

## 2. Materials and Methods

### 2.1. Data Analysis

For the purpose of the study, 1160 urgent referrals and the results of the head CT examinations were analyzed retrospectively, including CT of the head without contrast (C−), with intravenous (i.v.) contrast (C+) and angio-CT. Referrals were issued by the Emergency Department of the University Clinical Center of the Medical University of Silesia in Katowice between January and June 2021.

All data were collected and anonymized by the employees of the Department of Radiology and Nuclear Medicine of the Medical University of Silesia in Katowice.

The following parameters were included in the analysis: sex, age, type of examination (C−, C+, angio-CT), the content of the referral and the result of CT examination.

All data were collected in a specially prepared spreadsheet in Microsoft Excel, while the statistical analysis was carried out using Statistica 13.3. Statistical significance was established at *p* < 0.05.

### 2.2. Analysis of the CT Results

The result of the CT of the head was analyzed in each patient. Each of the pathologies identified by the radiologist was classified into the appropriate class from I to IV, according to the following key: Class I—no abnormalities were found in the imaging examination; Class II—included chronic lesions that did not cause symptoms (minor cortical atrophy, chronic vascular lesions and atherosclerosis); Class III—chronic, symptomatic changes (large cortical atrophy, large post-stroke lesions, painting cavities); Class IV—acute, symptomatic changes (ischemic stroke, hemorrhagic stroke and other forms of intracranial bleeding, tumor or injury).

### 2.3. Analysis of the Diagnostic Focus

The data from the referral were considered in terms of diagnostic orientation by the ordering party. Each diagnostic focus has been assigned to one out of seven categories, created based on the referral content and CT findings, as follows: Category 0—no targeting; Category 1—ischemic stroke/TIA; Category 2—hemorrhagic stroke or other intracranial bleeding; Category 3—tumor, metastases; Category 4—venous thrombosis; Category 5—epilepsy; Category 6—migraine.

### 2.4. Follow Up Examination with Contrast Enhancement

A total of 83 patients from the study group had two examinations performed while being diagnosed in the Emergency Department, including initial CT without contrast and complementary CT with contrast or angio-CT. The analysis was performed to determine the effect of the follow-up examination with i.v. contrast (CT C+, angio-CT) on the final diagnosis. The change in the size of individual classes between the initial and follow-up studies was assessed.

### 2.5. Presence of Symptoms

With regard to the content of the referrals, it was assessed whether the ordering party described the symptoms reported by the patient. Referrals with no description of the patient’s symptoms were assigned with 0, while those including symptoms in the description were assigned with 1.

### 2.6. The Analysis of the Convergence of the Diagnostic Orientation with the Results

In order to help assess the validity of the CT scans performed, we examined how often the targeted diagnostic tests coincided with the results confirming the presence of an acute condition (Class IV). The analysis included untargeted studies (Category 0) and targeted studies from Categories 1–4, excluding Category 5—epilepsy and Category 6—migraine, due to the lack of characteristic features allowing the diagnosis of these conditions in the CT examination. Follow-up examinations were also excluded from the analysis. Ultimately, 829 CT examinations without contrast enhancement were analyzed. Studies without and with a specified diagnostic target were then compared depending on the class of the obtained result (I–IV).

## 3. Results

### 3.1. Basic Data Analysis

In total, 1160 referrals were analyzed retrospectively, of which 594 referrals (51.21%) were issued for women and 565 (48.71%) for men. In one case, the gender of a patient with undetermined identity was not specified (0.09%)—this referral was excluded from the analysis.

The mean age of the patients was 62.78 years (+/−18.89), and there was no significant difference in the mean age between men and women (*p* = 0.258).

The most frequently chosen method of head imaging was CT without contrast (C−), which was ordered in 857 patients (73.94%). Contrast-enhanced CT (C+) and angio-CT examinations were performed in the same quantity. The studies differed in the gender and age distribution, as shown in Table 1.

The Kruskal–Wallis test was used to compare the significance of the mean age with the types of examinations. The mean age of patients undergoing non-contrast CT was higher and significantly different from the patients undergoing the same procedure with contrast enhancement (*p* < 0.01). It was also shown that the mean age of patients undergoing angio-CT was higher and significantly different from the mean age of patients undergoing contrast-enhanced CT (*p* = 0.08). There was no significant difference in the mean age of patients undergoing non-contrast CT and angio-CT examinations (*p* = 0.6).

### 3.2. Analysis of the CT Results

**Figure 1:**
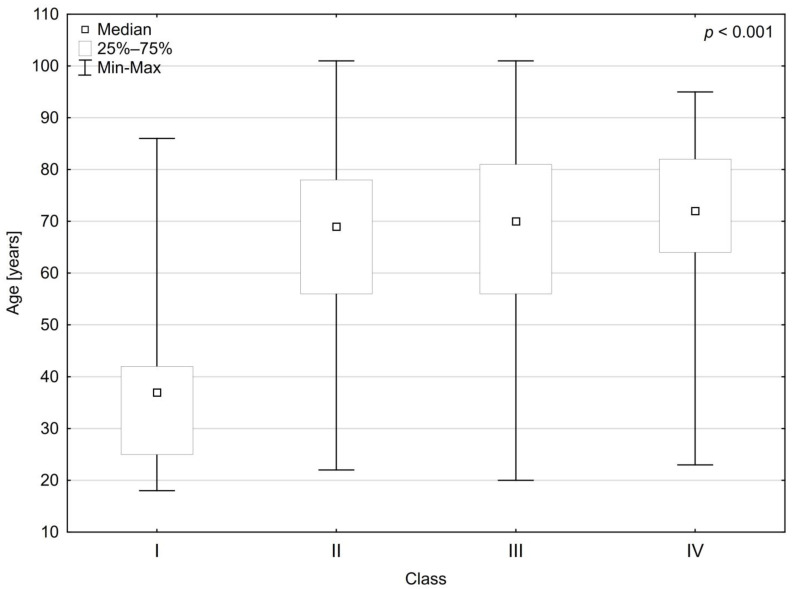
The differences in mean age of the patients depending on the class of computed tomography results.

Based on the analysis of CT examinations, results were assigned to four classes. The classes of CT results differed in age and gender distribution. An increase in the number of men and older adults in subsequent classes was noticed (Table 2, Figure 1).

The mean age of the patients with Class I results was lower and significantly different from the mean age of the patients in the other classes (II–IV) (*p* < 0.01). The mean age of the patients did not differ significantly in Classes II–IV.

**Figure 2:**
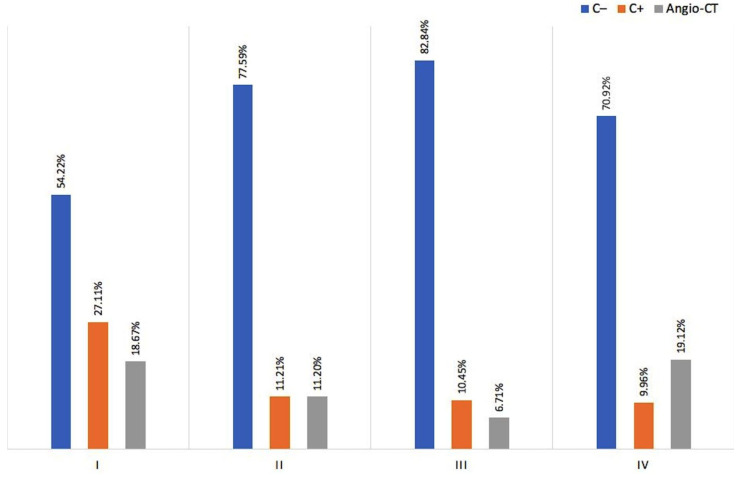
The dependence of the number and type of CT examinations on the classes of examination results (I–IV).

The study showed, using Pearson’s Chi^2^ test, that the number of patients in classes I–IV differed significantly depending on the examination performed (*p* < 0.01). CT without contrast (C−) was most frequently issued in patients with class II (chronic asymptomatic) and III (chronic symptomatic) findings, CT with i.v. contrast was most frequently issued in patients without any findings, whereas angio-CT was the most common examination in both Class I and IV (Figure 2).

### 3.3. Analysis of the Diagnostic Focus

The orientation of diagnostics on referral was assessed by categories (0–6). It was shown that as many as 855 referrals (73.71%) constituted Category 0, with a lack of diagnostic targeting of a specific pathology (Table 3).

In the analysis showing the dependence of the age of patients on the categories defining the orientation of diagnostics, it was found that the age of patients whose diagnosis was focused on ischemic stroke/TIA (Category 1) was higher and significantly different from patients whose diagnostic focus was classified into the remaining categories (0 and 2–6). The mean age of the patients among the other categories did not differ significantly.

### 3.4. Follow-Up Examination with Contrast Enhancement

**Figure 3:**
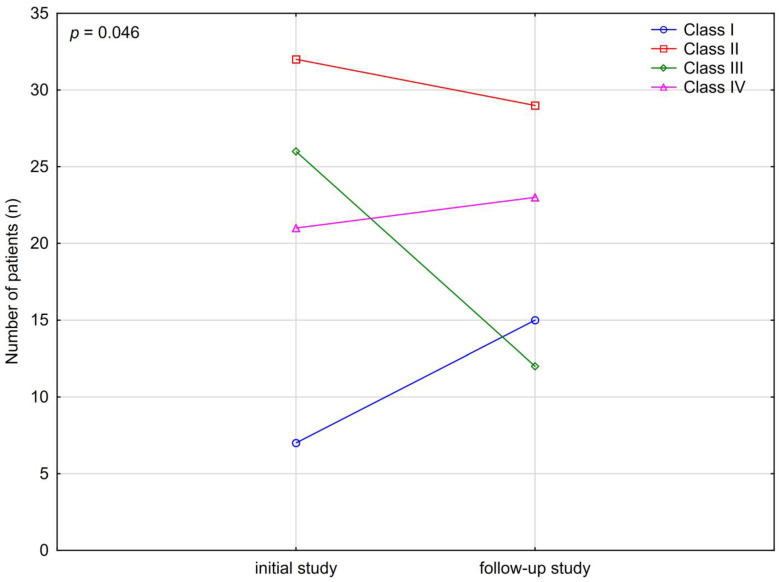
The analysis of the effect of the follow-up examination with i.v. contrast (CT C+, angio-CT) on the final diagnosis.

The effect of performing the follow-up examination with i.v. contrast (CT C+, angio-CT) after initial CT C− on the final diagnosis was analyzed in 166 referrals from 83 patients (Figure 3). Performing supplementary, contrast-enhanced examination allowed us to exclude the majority of Class III results characterizing chronic symptomatic lesions, favoring Class I results. The possible bias causing an increase in the results without changes (Class I) instead of the results describing non-significant chronic changes (Class 2) was due to the omission of the description of chronic changes during follow-up, which were described previously in the initial study. The increase in the number of Class IV (acute) results was statistically insignificant.

### 3.5. Presence of Symptoms

Referrals without description of symptoms accounted only for 7.07% (82). This could not be clearly demonstrated in the case of 23 referrals (1.98%), due to the subjective nature of these referrals. There were no statistically significant differences between the classes of results for referrals with and without symptoms.

### 3.6. The Analysis of the Convergence of the Diagnostic Orientation with the Results

In total, 649 out of 829 examinations (78.29%) had no diagnostic orientation specified on the referral. The remaining 180 examinations (21.72%) were targeted at one of four acute clinical conditions: ischemic stroke/TIA, hemorrhagic stroke or other intracranial bleeding, tumor or metastases and venous thrombosis (Table 4).

**Figure 4:**
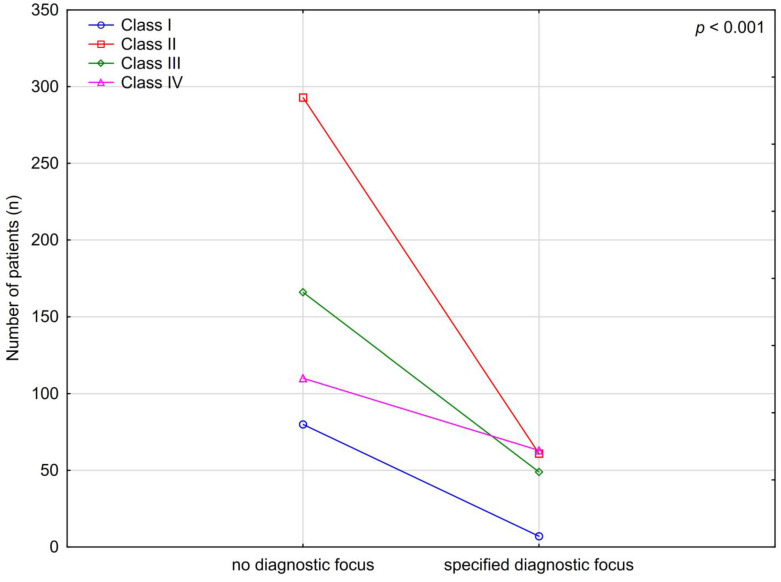
The analysis of the convergence of the presence of diagnostic orientation with the results of an initial head CT examination without contrast enhancement.

The analysis of grouped results showed that targeted referrals significantly more often coincided with the results from Class IV (acute conditions) than non-targeted referrals (Figure 4).

## 4. Discussion

The number of imaging examinations is constantly growing. In our previous study, the last decade saw a nearly two-fold increase in the number of CT scans performed [12]. Reasons behind this may be the greater demand for diagnostic imaging, especially in oncology, as well as the greater availability of imaging examinations due to modernization and the growing number of radiology departments. The high availability of CT facilitates diagnostic process, but, on the other hand, results in a greater workload as well as increasing population exposure to X-rays. Another harmful effect of too much access to diagnostic imaging is the issuing of unjustified referrals, which generate healthcare costs and extend the waiting time for patients who are truly in need of imaging.

A non-contrast head CT was the most frequently ordered examination regarding the urgent diagnostics of the central nervous system (CNS), which represented 73.94% of all tests. Meanwhile, over half (55.22%) of head CT scans did not show any abnormalities or clinically significant chronic changes. Patients representing Class I were predominantly younger than those assigned to the other classes (mean age 36.75 ± 11.74). Most of these patients presented mild complaints such as headache, nausea, dizziness or tinnitus. This indicates that the neurological symptoms reported by people of a younger age rarely have an organic background.

Acute conditions within the CNS were diagnosed in approximately one fifth (21.66%) of the patients. Along with the increasing number and severity of pathologies diagnosed in the CNS, the percentage of male patients increased as well (42.17% within the first class and 55.78% in the fourth class). This parameter could support the current reports of stronger hypochondriac trends in women, who often exaggerate their symptoms in order to be admitted to the hospital for no specific reason [27,28].

We have shown that the presence of specific clinical targeting on a referral for CT head examination significantly correlates with a higher frequency of acute diagnosis. This means that referrals issued to confirm clinical suspicions are more accurate and therefore more justified than those issued to exclude undefined pathologies. This observation may result from a more in-depth examination of patients and a more precise issuing of referrals, but also more obvious symptoms presented by patients with acute neurological conditions. Unfortunately, 73.71% of the analyzed referrals did not include any diagnostic target or any diagnostic suggestion, and although the presence of symptoms reported by the patient was included in more than 90% of referrals, most were not very suggestive or were given in a laconic manner, which reflects the negligence of physicians when referring patients for diagnostic imaging [29].

Another attention-drawing matter was migraine, marked in the sixth category of reasons behind performing a CT scan. It is widely known that the main diagnostic instrument to examine this condition is magnetic resonance imaging (MRI). Head CT may be ordered in case of a drug-resistant headache and in the search of brain tumors; however, it should be performed with i.v. contrast enhancement. Such findings may lead to the conclusion that the above-mentioned patients, constituting 2.16% of all patients, were sent to an inappropriate examination, which stands against the official guidelines, probably in order to meet the patients’ expectations and, at the same time, to reduce the costs by choosing a cheaper and faster plain CT scan instead of contrast-enhanced CT or MRI.

A significant observation was made that supplementary, contrast-enhanced examination allowed the exclusion of the majority of Class III results characterizing chronic symptomatic lesions. This indicates that contrast-enhanced follow-up examinations allowed the unequivocal classification of patients into extreme classes (I or IV) and accurate identification of patients requiring urgent or chronic treatment. The question remains as to whether the follow-up examination had a significant influence on therapeutic decisions, which would justify an additional dose of radiation and i.v. contrast administration. In our study, CT with contrast more often excluded acute pathology than confirmed it. Chishti et al. found that contrast-enhanced head CT performed after non-enhanced CT showed new lesions and changed the initial diagnosis in merely 0.5% and 2.7% of patients, respectively [30]. By following the relevant recommendations, the authors proved that only 9.7% of patients would benefit from an additional contrast-enhanced head CT.

According to a survey of Spanish general practitioners (GPs), pediatricians and nurses, intrusive patient requests are responsible for more than 60% of the misuse of prescriptions, referrals for diagnostic examinations or treatments. Despite the fact that communication strategies with the demanding patient have been described for years, this study showed a number of reasons for its failure—apart from pressure from the patient, there was also a lack of time for a proper consultation, the desire to gain greater safety and control over the case and the desire to satisfy the patient or avoid their claims. Interestingly, 28.1% of GPs admitted that they still do not know how to make the patient understand that a given procedure is unnecessary [28]. In one of the studies, it has been shown that merely 7% of patients referred for diagnostic imaging receive any information about the exposure to radiation [31]. Other research reveals the constant dispute over which specialist should provide the patient with such information, either the primary care physician or a radiologist. These figures may be disturbing, as they uncover the low awareness about the possible effects of radiation exposure, while this may affect the patient’s final decision as to whether or not to give consent for the procedure. The doctors’ lack of assertiveness and the poor ability to communicate properly with the patient for the sake of their health is disappointing [32,33]. The absence of these skills and the extremely short time of the appointment make it almost impossible to change the patients’ pattern of perceiving obtaining a head CT referral as superior to inevitable exposure to radiation. It should also be pointed out that the daily dose of absorbed background radiation is 0.007–0.008 mSv, whereas a routine head CT scan entails absorption of 1.8–2.8 mSv, which represents a 350-times larger number than the regular daily dose of radiation [34,35]. This figure may be important for patients who either suffer from severe illnesses such as tumors or, regarding their young age, are planning to start a family, as the high doses of radiation may affect fertility [36]. Our results mostly showed no abnormalities in the youngest age group. Such demanding behavior among patients is possibly associated with the growing number of anxiety disorders in the population and the tendency for somatization; therefore, a thorough, careful interview with each patient, which includes a psychiatric aspect, is necessary [37]. Following the diagnostic guidelines should also decrease the scale of the discussed problems. Considering that healthcare systems are usually underfunded, it is worth paying attention to the costs generated by performing unjustified imaging tests [38]. Among the studied group, 14.34% did not show any abnormalities in their CT scan. According to Statistics Poland, in 2016, there were approximately 1,871,600 CT scans performed in Poland [39]. Assuming that the similar percentage of these examinations as in our study did not show any abnormalities, at least 262,024 such scans were most likely issued and performed unnecessarily. Therefore, it seems obvious that promoting the responsible commissioning of imaging examinations will contribute to a significant drop in healthcare costs.

It is difficult to objectively assess the rationale for ordering imaging examinations, as important information from the medical history, results of physical examinations and additional tests are often not included in referrals to the radiology department. Moreover, a negative imaging study does not necessarily constitute a defensive approach or misuse. Imaging examinations should be performed based on clinical indications, in order to confirm a certain pathology rather than to exclude it.

## 5. Conclusions

Over half of the results of urgent head CTs showed no or chronic non-significant lesions. Contrast-enhanced follow-up examinations more often excluded acute pathology than confirmed it. These observations may indicate that the head CT procedures are highly overused in an acute setting. The presence of specific clinical targeting on a referral for CT head examination significantly correlates with a higher frequency of an acute diagnosis. A discussion with medical professionals ordering CT examinations as a matter of urgency seems essential and inevitable, since they are generating tremendous costs and exposing patients to radiation and contrast. The emphasis should be placed on improving the content of referrals, as well as the communication between physicians and their patients, particularly those with hypochondriac tendencies.

## Figures and Tables

**Table 1:** The characteristics of patients depending on the type of examination ordered.

Type of Examination	Number of Patients			Mean Age	*p*
	Men, 565 (48.75%)	Women, 594 (51.25%)	All, *N* (%)		
CT C−	437 (77.35)	420 (70.7)	857 (73.94)	62.23 ± 18.54	0.968
CT C+	69 (12.21)	82 (13.8)	151 (13.03%)	55.23 ± 19.63	0.051
angio-CT	59 (10.44)	92 (15.5)	151 (13.03%)	62.23 ± 18.34	0.483

CT—computed tomography; C−—without contrast; C+—with i.v. contrast.

**Table 2:** The characteristics of patients depending on the class of computed tomography results.

Class	Number of Patients			Mean Age	*p*
	Men, 565 (48.75%)	Women, 594 (51.25%)	All, *N* (%)		
I	70 (12.39)	96 (16.16)	166 (14.32)	36.75 ± 11.74	0.691
II	227 (40.18)	247 (41.58)	474 (40.9)	66.07 ± 16.07	0.509
III	128 (20.65)	140 (23.57)	268 (23.12)	66.59 ± 17.78	0.552
IV	140 (24.78)	111 (18.67)	251 (21.66)	69.64 ± 14.19	0.683

Classes: I—no changes; II—chronic asymptomatic lesions; III—chronic symptomatic lesions; IV—acute lesions.

**Table 3:** The characteristics of patients depending on the category of diagnostic focus.

Category	Number of Patients			Mean Age	*p*
	Men, 565 (48.75%)	Women, 594 (51.25%)	All, *N* (%)		
0	418 (74.0)	437 (73.57)	855 (73.71)	61.68 ± 19.31	0.087
1	81 (14.34)	95 (16.0)	176 (15.17)	71.69 ± 13.53	0.995
2	31 (5.48)	24 (4.04)	55 (4.74)	62.71 ± 16.94	0.194
3	12 (2.12)	10 (1.68)	22 (1.90)	53.91 ± 15.69	0.869
4	8 (1.41)	5 (0.84)	13 (1.12)	48.15 ± 21.10	0.607
5	6 (1.06)	7 (1.18)	13 (1.12)	53.54 ± 21.02	0.224
6	9 (1.59)	16 (2.69)	25 (2.16)	54.44 ± 20.52	0.335

Categories: 0—no targeting; 1—ischemic stroke/TIA; 2—hemorrhagic stroke or other intracranial bleeding; 3—tumor, metastases; 4—venous thrombosis; 5—epilepsy; 6—migraine.

**Table 4:** The analysis of the convergence of the diagnostic orientation with the class of the results of an initial head CT examination without contrast enhancement.

Class of the Result	Orientation Category					
	0, 649 (78.29%)	1, 135 (16.29%)	2, 37 (4.46%)	3, 5 (0.6%)	4, 3 (0.36%)	All, *N* (%)
I	80 (12.32)	3 (2.22)	3 (8.11)	1 (20.0)	0 (0)	87 (10.5)
II	293 (45.15)	51 (37.78)	8 (21.621)	1 (20.0)	1 (33.33)	354 (42.7)
III	166 (25.58)	36 (26.67)	11 (29.73)	2 (40.0)	0 (0)	215 (25.93)
IV	110 (16.95)	45 (33.33)	15 (40.54)	1 (20.0)	2 (66.67)	173 (20.87)

Classes: I—no changes; II—chronic asymptomatic lesions; III—chronic symptomatic lesions; IV—acute lesions. Categories: 0—no targeting; 1—ischemic stroke/TIA; 2—hemorrhagic stroke or other intracranial bleeding; 3—tumor, metastases; 4—venous thrombosis.

## Data Availability

Not applicable.
